# Risk indices that predict in-hospital mortality of elderly patients

**DOI:** 10.3906/sag-2005-67

**Published:** 2020-06-23

**Authors:** Dilek DÜLGER, Özgür ALBUZ

**Affiliations:** 1 Department of Microbiology, Faculty of Medicine, Karabük University, Karabük Turkey; 2 Deparment of General Surgery, Keçiören Training and Research Hospital,Ankara Turkey

**Keywords:** Elderly, follow-up care, mortality, risk factor

## Abstract

**Background/aim:**

Mortality in the elderly population tends to be higher than in all other age groups; the risk factors that predict mortality among those in this age cohort are not fully understood. This large-scale clinical study aimed to identify effective risk factors that predict mortality in the elderly population with a particular focus on age and hospitalization status.

**Material and methods:**

We retrospectively analyzed outcomes from patients with clinical follow-up between July 2015 and January 2020 at 29 Mayıs State Hospital, Ankara, Turkey. Patient records with missing or ambiguous data were excluded. Age, sex, length of hospital stay, comorbidities, consultation requests and diagnoses that include infectious diseases were evaluated for their role in predicting in-hospital mortality using binary logistic regression analysis. Primary outcomes focused on factors that had an impact on overall in-hospital mortality in the elderly population.

**Results:**

Our study included 11,430 patients; of this group, 39.9% were elderly, which we defined as 65 years of age or older. Risk factors for in-hospital mortality in this cohort included consultation requests (AOR = 1.95, CI (1.53–2.49), P < 0.001) and length of hospital stay of ≥4 days (AOR = 2.49, CI (1.90–3.26), P < 0.001).

**Conclusion:**

Elderly patients are at significantly higher risk for in-hospital mortality than are younger patients. Among the factors that may be used to predict the risk of in-hospital mortality in the elderly patient cohort, the most important factor is the length of hospital stay.

## 1. Introduction

Mortality tends to be higher in elderly patients compared to younger patients in both in-hospital and outpatient settings across all fields of medicine; this is in spite of the that improved living conditions and medical advances have led to steady increases in the life span [1–3]. Hospitalization is associated with unique risk factors, as patients may encounter different stresses that ultimately complicate morbidity and lead to mortality. In this study, our goal was to determine what factors were specifically associated with and might be used to predict an increased risk of in-hospital mortality in an elderly patient cohort. Identification of one or more risk factors for in-hospital mortality may provide information on ways to intervene in order to minimize this outcome; however, risk factors that serve as predictive measures alone will also be of value. Identification of factors associated with in-hospital mortality may help the hospital staff to anticipate problems and to identify individuals in need of particularly attention and care. In literature, there are not too many studies that have clearly identified factors that predict mortality in elderly patients presenting at outpatient clinics. On the other hand an understanding of the factors that predict mortality will be essential for the appropriate management of patients undergoing clinical follow-up by healthcare professionals. As such, in this study we have evaluated several prominent variables associated with mortality among elderly patients who were admitted to our state hospital and we identified specific factors that may be useful for prediction of mortality within this patient cohort. 

## 2. Materials and methods

### 2.1. Patients

**Figure 1:**
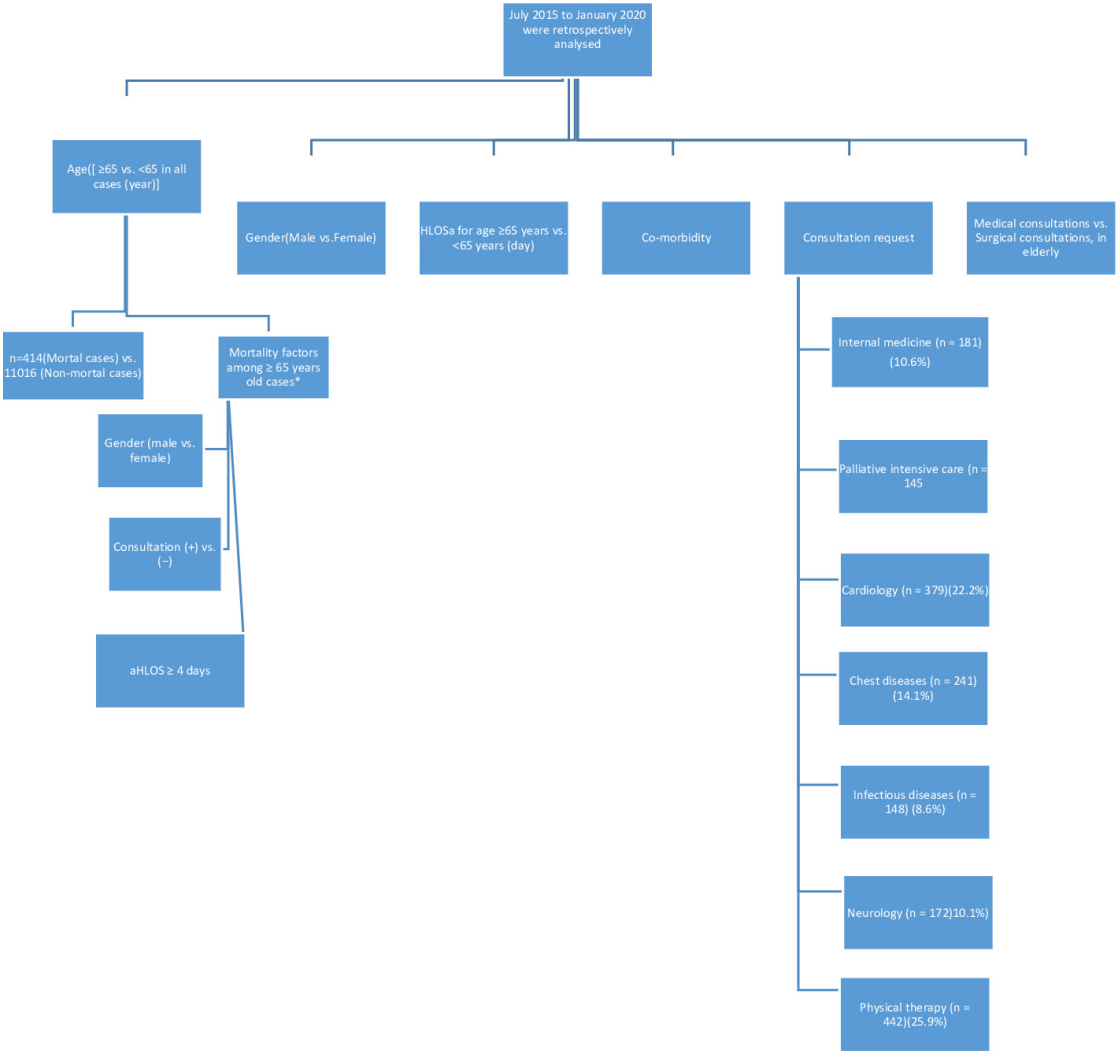
Patient flow chart.

This retrospective study was approved on January 16, 2020 by the Ankara City Hospital Ethics Committee of the Ministry of Health Provincial Health Directorate (approval number of E1-20-263). Patients were identified via a retrospective examination of the registry entries of 29 Mayıs State Hospital from July 2015 through January 2020. A total of 11,720 patients were admitted to our state hospital during this interval. Patients with unclear diagnoses and/or incomplete records were excluded from the study; 11,430 cases were included in the evaluation. Data collected from each patient case were as follows: age, sex, length of hospital stay (LOHS), the use of clinical consultation, surgical vs. medical comorbidities, diagnosis of infectious and mortality during the follow-up period. The primary outcome measure was in-hospital mortality. As part of our analysis, we identified patients who did and who did not die which hospitalized, and collected information on demographics, LOHS, distribution of clinical consultations requested for the elderly patients, and patterns medical vs. surgical consultations. Given the retrospective design of this study, the clinical follow-up period for each patient was determined by the LOHS (Figure 1).

### 2.2. Statistical analysis

Data for dichotomous variables are presented as frequencies and percentages; continuous variables are presented as mean ±standard deviation. We used the Kolmogorov–Smirnov test to identify normal distributions. In the univariate analysis of the variables associated with mortality, Student’s t-tests or Mann Whitney U tests were used to evaluate the continuous variables according to the nature of the distribution; chi-square tests were used to evaluate binary variables. The level of significance (α) was set at 0.05. Linear stepwise and binary backward logistic regression modeling were used to identify the impact of selected outcomes on the elderly population after adjusting for differences in age, sex, LOHS, and consultation status. In addition, the test compatibility of the backward stepwise binary logistic regression was used with the Hosmer-Lemeshow and Omnibus P-value association tests (OPATs) tests in order to determine whether the variance in the dataset was significantly larger than the unexplained variance. The statistically significant variables were then included in the binary logistic regression analysis and the area under the curve (AUC) was calculated for each independent risk factor. The adjusted odds ratios (AORs) and AUC were examined using receiver operating characteristic (ROC) curve analysis. Sensitivity and specificity were analyzed with SPSS™ 18.0 for Windows (SPSS, Chicago, IL, USA). 

## 3. Results

### 3.1. Patient characteristics

This study included clinical follow-up of 11,328 patients of whom 4523 (39.9%) were ≥65 years of age. The mean ages of patients older and younger than 65 years of age were 74.89 ± 7.36 and 48.29 ± 12.29 years, respectively (P < 0.001). The LOHS for all patients was 15.72 ± 8.79 days with no significant differences observed among patients older or younger than 65 years of age; median LOHS [2 day, (IQR: 3) vs. 2 day, (IQR: 3) ; P = 0.316)]. Female and male patients accounted for 1486 (59.8%) and 997 (40.2%), respectively, of the total patient cohort (P < 0.001); the mean ages of female and male patients were 57.19 ± 17.59 and 58.48 ± 17.57 years, respectively (P = 0.06). The descriptive variables, for distribution of clinical comorbidities, are included in Tables 1 and 2.

**Table 1:** Clinical services and comorbidities of all hospitalized patients in the surgical clinics.

General surgeryn = 2876 (25.2%)	Urologyn = 690 (6%)	Orthopedics and traumatologyn = 1637 (14.3%)	Cardiovasculary surgeryn = 627 (5.5%)
Inguinal hernia (n = 922) Abdominal hernia (n = 125) Umbilical hernia (n = 156) Other hernias (n = 9) Gastroesophageal reflux (n = 1281) Peptic ulcer (n = 1341) Appendicitis (n = 168) Diverticulitis (n = 4) Pelvic inflammatory disease (n = 18) Cholelithiasis (n = 1018) Soft tissue infection (n = 97) Skin abscess (n = 75) Acute cholecystitis (n = 11) Anorectal abscess (n = 3) Decubitus ulcer (n =75) Malignancy (n = 118) Hemorrhoids (n = 70) Fecal incontinence (n = 85) Decubitus ulcer (n = 75)	BPH (n = 512) U. incontinence (n = 114) Cystitis (n = 67) Hematuria (n = 88)	Osteomyelitis-osteonecrosis (n = 5) Extremity fractures (5) Cellulitis (n = 107) Thrombophlebitis (n = 382)	Cardiac failure (n = 19) Aortic dissection (n = 6) Pulmonary embolism (n = 51) Venous thromboembolism (n = 32) Acute cardiac ischemic disease (n = 15) Vascular aneurysm (n = 6)

All comorbidities were stratified according to clinics.

**Table 2:** Clinical services and comorbidities of all hospitalized patients in other clinics.

Cardiology n = 547 (4.8%)	Chest diseases n = 243 (%2.1)	Internal medicine n = 1018 (8.9%)	Coronary intensive care n = 1657 (14.5%)	Internal intensive care n = 926 (8.1%)	Palliative care unit n = 247 (2.2%)
EPH (n = 429) Cardiac failure (n = 79) Hypotension (n = 39)	A.bronchitis (n = 96) C. bronchitis (n = 17) Pulmonary edema (n = 15) Pneumonia (n = 164) PNX (n = 10)	EPH (n = 995) Hypothyroidism (n = 24) Pancreatic disease (n = 7)	A.pericarditis (n = 6) Atrial fibrillation (n = 214) AMI (n = 497) EPH (n = 601)	Type I DM (n = 72) Type II DM (n = 345) ARF and CRF (n = 137)	Alzheimer (n = 46) Parkinson (n = 34) Delirium (n = 2) TIA (n = 7) Subarachnoid hemorrhage (n = 5) ICII (n = 73) Oliguria-FEI (n = 6)

All comorbidities were stratified according to clinics.ICII: Intracerebral infarction/ischemia, P.D: Pancreatic disease, C: Chronic, A: Acute, TIA: Transient ischemic attack, U: Urinary, ARF: Acute renal failure, DM: Diabetes mellitus, CRF: Chronic renal failure, BPH: Benign prostatic hyperplasia, PNX: Pneumothorax, EPH: Essential primary hypertension,

We also evaluated the types of infections reported for these patients. We found that 21 patients were diagnosed with sepsis, 16 had pneumonia and the 10 remaining had soft tissue infections associated with severe diabetic foot and decubitus ulcers. Distribution of cases in hospital clinical mortality were found general intensive care 274 (66.2%), palliative care unit 110 (26.5%), cardiovascular and cardiovascular intensive care unit 26 (6.2%), orthopedics and trauma 3 (1.45%), neurology 1 (0.48%). The highest rates of in-hospital mortality were associated with the internal medicine services; of these, general intensive care units experienced the highest rate of in-hospital mortality.

### 3.2. Factors associated with mortality

Tables 3 and 4 summarize the results of univariate analysis of factors associated with in-hospital mortality. We found that, of the 2,485 patients who sought and received consultation (+), 235 (9.5%) ultimately died while in the hospital. Among those patients who received consultations, 1564 (87.8%) were ≥65 years of age and 908 (12.2%) were <65 years of age (P < 0.001). Of the 414 in-hospital mortality cases in this study, 338 patients were ≥65 years of age (81.6%; P < 0.001). Of the patients who died while remaining in the hospital, 298 (72%) had a LOHS of ≥4 days; this was statistically significant compared with the number of patients who died in the hospital after a LOHS of <4 days (28%; P < 0.001). Sex was not found to be a significant risk factor for mortality (Table3).

**Table 3:** Results of univariate analysis: potential risk factors among patients that died in the hospital.

	Age ≥65 vs. <65 years	Sex (male vs. female)	Consultation (+) vs. (−)	aLOHS ≥ 4 days	Comorbidity
Mortality	338 (81.6%)/434(%100) vs.96 (18.4%)/434(%100)	213 (51.4%) /434(%100) vs.201 (48.6%)/434(100%)	235 (67.4%)/434(100%) vs.179 (32.6%)/434(100%)	298 (72.0%)/434(100%) vs.116 (28.00%)/434(100%)	314(71.9%)/434(100%) vs.123(28.1%)/434(100%)
P value <0.05	0.001	0.114	0.001	0.001	0.147

aLength of hospital stay.

**Table 4:** Results of univariate analysis: patients ≥65 years of age who died while in the hospital.

	Factors contributing to mortality among those ≥ 65 years of age; *n = 337 vs. 4523	P < 0.05
Sex (male vs. female)	164(3.63%)/4523 vs. 173(3.82%)/4523	0.11
Comorbidity (+) vs. (-)	249(7.4%)/3343 vs. 89(7.5%)/1180	0.916
Consultation (+) vs. (−)	[196(12.5%)/1368(87.5%)] vs. [141(4.8%)/ 2813(95.2%)]	0.001
aLOHS ≥ 4 days	[247 (11.6%) vs.1892 (88.4%) (between mortal cases)]	0.001
	[91(3.8%) vs. 2288 (96.2%) (between nonmortal cases)]	

aLength of hospital stay.

All significant variables were included in binary logistic regression analysis for mortality among those aged ≥65 years and those aged <65 years. With this analysis, we determined that age ≥65 years of age, LOSH and consultation (+) remained significant risk factors (P < 0.001 for all), with AOR (95% confidence interval (CI) of 4.54 (3.50–5.89), 2.86 (2.24–3.65) and 2.24 (1.79–2.80), respectively (Table 5). Additionally, when we evaluated mortality among only those over than 65 years of age, the following results were obtained: AOR (CI) for consultation (+) [AOR: 1.95 (1.53–2.49; P < 0.001)] and for LOHS ≥4 days [AOR: 2.49 (1.90–3.26; P < 0.001)] (Table 5).

**Table 5:** Outcomes associated with in-hospital mortality.

Predictors of in-hospital mortality for patients <65 and ≥ 65 years old		*Adjusted odds ratio (95% CI)/Adjusted mean difference (95% CI) for Exp (B)		
	Sig	Exp (B)	Lower	Upper
Consultation (+) vs. (−)	0.001	2.24	1.79	2.80
LOHS (day)	0.001	2.86	2.24	3.65
Age ≥65 vs.<65 (year)	0.001	4.54	3.50	5.89
Comorbidity	0.698	0.956	0.76	1.20
Predictors of in-hospital mortality for those ≥ 65 years old; mortalityvs. nonmortality among those in the elderly patient cohort.		*Adjusted odds ratio (95% CI)/Adjusted mean difference (95% CI) for Exp (B)		
	Sig	Exp (B)	Lower	Upper
Consultation (+) vs. (−)	0.001	1.95	1.53	2.49
HLOS (day)	0.001	2.49	1,9	3.26

*Linear stepwise and binary backward logistic regression analyses adjusting for differences in age, sex, LOHS, consultation status.

###  3.3. Calculated AUC results with ROC curve analysis including the risks indexed for each patient. 

**Figure 2:**
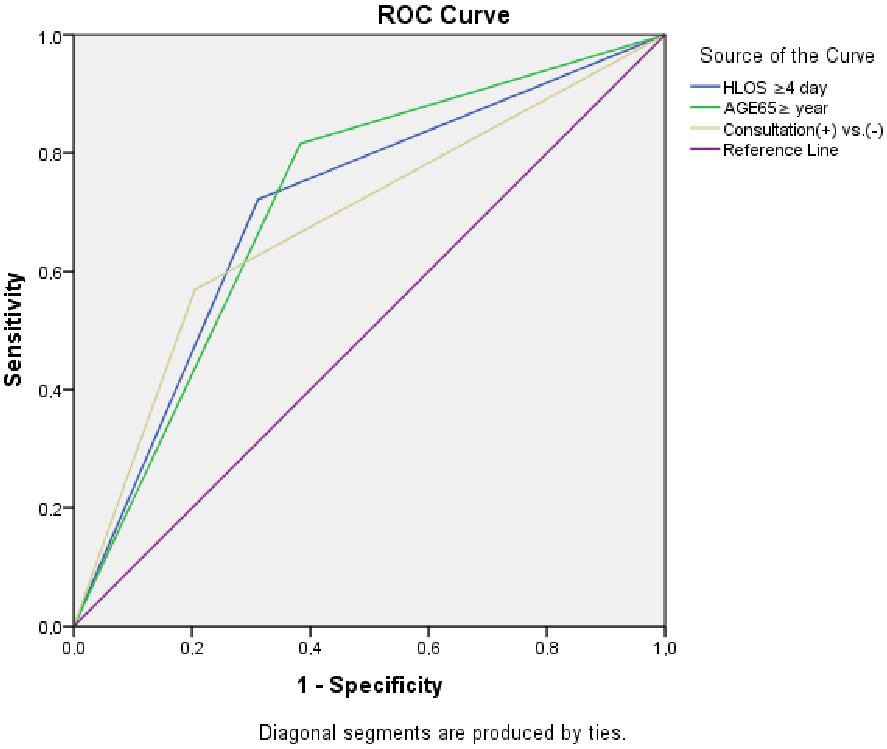
Area under curve (AUC) of the receiver operator characteristic (ROC) curve with respect to factors predicting in-hospital mortality.

Adjusted mortality risk ratios for those who died while in the hospital versus those who did not were calculated for patient consultations using ROC analysis (Figure 2); a significant difference (P < 0.001) was identified. Consultation, (+) vs. (−); LOHS, ≥4 days vs. <4 days and age, ≥65 years vs. <65years resulted in AUC values (95% CI) of 0.682 (0.654, 0.711), 0.705 (0.680, 0.730) and 0.716 (0.693, 0.739), respectively (Table 6; Figure 2).

**Table 6:** Comparisons among the significant predictors of mortality.

			*Asymptotic 95% Confidence interval (95% CI) for AUC		Positive if greater than or equal toa	Sensitivity, specificity, predictive values	
Mortality vs. Nonmortality(n = 11430)	Sig	AUC					
Consultation (+) vs. (−)	0.001	0.682	0.654	0.711	,5000*	0.576	0.327
LOHS (day)	0.001	0.705	0.680	0.730	,5000*	,730	,453
Age ≥65 vs. <65 (year)	0.001	0.716	0.693	0.739	,0000*	1,000	1,000

a*: The positive actual state is 1.00.Assessment was performed using ROC curve analysis for total cases together with specific results associated with the elderly patient cohort.

The receiver operating characteristic (ROC) curve was used here to generate a sensitivity to predictive ratio in cases in which the separation threshold value differs in binary classification systems. LOHS, ≥4 days was associated with the highest area under curve.

## 4. Discussion

Due to a comparatively low birth rate and increased life expectancy, the proportion of the population defined as elderly is growing rapidly in the United States and worldwide. Of the estimated 7.3 billion people worldwide in 2015, 617.1 million (9%) were at least 65 years of age [4]. In Turkey, the elderly population is predicted to reach 17.6% by 2050 [5]. Elderly people often have chronic diseases and are at a greater risk of experiencing complications associated with hospitalization in internal medicine clinics as well as during surgery. In this study, we focused on a clinical follow-up of a hospitalized elderly cohort. We searched numerous indices in order to identify risk factors that would predict mortality in this population. The most prominent risk factor associated with mortality is age ≥65 years. However, we believe that additional risk factors may be identified by evaluation of critical parameters in prospective, multicenter studies. In this study, risk factors that predict mortality were determined practically using data collected from a large number of clinical inpatients. Our goal was to identify straightforward and practical risk factors that would facilitate prediction of risk status by both the responsible physician and by other healthcare personnel. For example, systematic improvements in hospital service might serve to limit prolonged LOHS and may ultimately promote favorable outcomes; it is well known that prolonged LOHS results in a significant impact on muscle mass, functional capacity and risk of surgical complications [6–8].

Among the main comorbidities, the diagnosis of diabetes mellitus (DM) accounted for the highest number of cases (n = 417). Furthermore, 171 patients aged ≥65 years accounted for ~41.7% of all comorbidities identified. Notably, a considerable proportion of the infection-related surgical complications were identified among patients diagnosed with DM; this accounted for ~9% of all cases evaluated in this study. Taken together with the findings of Tang et al. [9], this result provides even further support for early and effective treatment of DM in order to prevent future significant complications. Approximately 15 years ago, Bouillanne et al. [10] introduced the geriatric nutrition risk index in order to assess the nutritional status of elderly populations and to measure nutrition-associated mortality risk. Carrero et al. [11] further reported that malnutrition was the most common complication among patients undergoing dialysis; nearly 28%–54% of patients on maintenance dialysis experience nutritional problems. In the present study, a diagnosis of DM correlated with acute or chronic renal failure (RF-positive, n = 89 vs. RF-negative, n = 66) and decubitus ulcers (DM-positive; n = 58 vs. DM-negative, n = 20). After univariate analysis; comorbidities were integrated into binary logistic regression analysis too.

However, no specific comorbidity predicted in-hospital mortality. As noted above, the most important factor associated with in-hospital mortality in our study was age ≥65 years. Interestingly, Maia et al. [12] noted that, as women live longer than men, chronic illnesses and comorbidities may have a more profound negatively impact on men for longer periods. Furthermore, poor personal health was more closely associated with the quality of life among women than among men while conversely, men’s perception of poor health was associated with an increased risk of mortality [9,12]. Similar to our findings, age was the best indicator of the mortality risk among the factors analyzed; this was attributed to the increased like lihood of acquiring a chronic disease or disability with increasing age [12]. Likewise, old age was identified by Byrne [13] and by Wolinsky et al. [14] as the most important factor associated with mortality; our findings are consistent with those previously reported. Similarly, Soong et al. [15] reported that increased the frailty is among the most prominent features observed among the elderly, and that reduction of the physiological reserve was frequently a factor associated with the aging process; the role of the Foster global vulnerability score as important for developing risk prediction models for hospitalized elderly [15]. We agree that the most important multifactorial term with a substantial impact on hospital mortality is fragility; we believe that the basis of fragility is the decrease in physiological reserves associated with aging.

The second most important factor associated with mortality in the present study was a LOHS of ≥4 days. Arnold et al. [16] reported that LOHS was directly associated with the risk of mortality from pneumonia among elderly patients. However, Ghassibia et al. [17] found no statistically significant associations between LOHS and mortality. Interestingly, we consider the results of a recent study [18] that included the formulation of an epidemiological profile and identification of main determinants of morbidity and mortality in patients considered to be at high risk for noncardiac surgery. Although the patients in this earlier study were in a high surgical risk group, LOHS and age were associated with more complications in our study; although not as prominent as the age, LOHS is one of the variables we identified that predict in-hospital mortality. We believe that LOHS inpatients with pelvic fractures were not significantly different from what was observed here; indeed, many of the patients in our study were already hospitalized for a long period of time due to pelvic fractures [17].

The third predictive factor that we identified as predictive of in-hospital mortality was the need for clinical consultation. We believe that the issue of consultations among clinical services may be a risk factor for mortality in the elderly population, as this may imply that issues that develop acutely or those that are noted and addressed later on may reflect on complexities that are not readily solved by one clinical service alone. It is clear that elderly patients should undergo careful study during the critical clinical follow-up period. Malak et al. [19] reported that data collection, consultation and the integration of numerous clinical components were important in managing patients in a neonatal ICU; this group also noted the importance of establishing an artificial intelligence system in the neonatal ICU with the goal of identifying predictive factors to be used in mortality estimates [19]. Likewise, Khan et al. [8] emphasized that collaborative and consultative geriatric care can improve the management of older surgical patients by potentially reducing the LOHS, identifying high-risk patients and facilitating early and appropriate specialty input in addition to outpatient follow-up. We believe that the relationship of medical consultations to in-hospital mortality may be directly related to the complexities associated with specific patient management. In other words, we do not consider consultations to be causative, but instead, they serve as a marker that reflects increased risk due to a higher level of complexity associated with the management of specific patients. In this sense, consultations may be a marker related to mortality and an indirect reflection of multiple risks. As highlighted in this study, this understanding coincides with the LOHS observed and the importance of consultation and age [8]. In the elderly population, adverse outcomes are predominantly associated with major surgery [20–22]. However, we note that, in the elderly population, medical consultations were requested more frequently than surgical consultations (1339 vs. 1040), although this difference did not reach statistical significance (P > 0.05). The most frequently consulted clinical services were physical therapy [(n = 442) (25.9%)], cardiology [(n = 379) (22.2%)], pulmonary [(n = 241) (14.1%)], internal medicine [(n = 181) (10.6%)], neurology [(n = 172) (10.1%)], infectious diseases [(n = 148) (8.6%)] and palliative intensive care [(n = 145) (8.5%)]. Martinez et al. [23] also emphasized that shorter LOHS will reduce various complications such as geriatric syndrome and the subsequent domino effect. In addition, they emphasized that improving the quality of healthcare for the elderly, particularly organization of transportation services, may provide better outcomes. According to our hypothesis, effective consultations and short LOHS would improve the outcomes of elderly patients more than among those who are younger.

Bruno et al. reported that intima-media thickening in large vessels became more evident from the 4th decade of life and vascular remodeling change related to essential hypertension occurred in the 50s [24]. In our study, we see that a total of 2082 patients with essential hypertension consisted of approximately 956 (45.9%) ​​of patients under 65 years of age. From this point of view, although we think that comorbidity is important for mortality; This effect decreases in terms of mortality due to the similar comorbidity prevalence among comorbid between mortal vs. nonmortal patients in our study group.

Similarly, patients with age 65 and above, Type I diabetes mellitus (DM), which is another comobidity factor among patients under 65, were 39 vs. 33 and Type II DM cases were 178 vs 149. Additionally among 11,500 cases, 18 of 41 patients diagnosed with acute renal failure had deceased and there was not found statistically significance (P > 0.05), in terms of acute renal failure effects over mortality, in our series. To determine valid and reliable methods for treatment strategies, an understanding of the risk of mortality remains critical. In this study, our aim was to identify risk factors associated with in-hospital mortality in an elderly patient cohort. These factors may constitute the first steps toward establishing a scoring system to be used to determine the risk of in-hospital mortality of elderly patients. However, this approach will need further development and research via a more comprehensive multicenter study. Nevertheless, our approach provides the first step towards classifying this most critical patient cohort. Although this research has been structured to encapsulate a wide spectrum of cases and a large body of clear and coherent data, the risk assessment remains somewhat limited due to the retrospective nature of the study design. 

In conclusion, among the risk factors associated with in-hospital mortality, the most important risk factor among those defined as elderly ( ≥65 years old ) is a LOHS ≥4 days; the second most important risk factor is the need for interservice clinical consultation. Compared to the younger group, age ≥65 years old stands alone as risk factor for in-hospital mortality. Taken together, these 3 indexes may predict mortality in a hospitalized elderly cohort. However, multicenter and prospective studies will necessary to validate and improve these predictive indices.
